# Prior exposure to inhaled allergen enhances anti-viral immunity and T cell priming by dendritic cells

**DOI:** 10.1371/journal.pone.0190063

**Published:** 2018-01-02

**Authors:** Debbie C. P. Lee, Neil Q. Tay, Marini Thian, Nayana Prabhu, Kazuki Furuhashi, David M. Kemeny

**Affiliations:** Immunology Programme, Department of Microbiology and Immunology, Centre for Life Sciences, National University of Singapore, Singapore; University of Michigan Health System, UNITED STATES

## Abstract

Influenza and asthma are two of the major public health concerns in the world today. During the 2009 influenza pandemic asthma was found to be the commonest comorbid illness of patients admitted to hospital. Unexpectedly, it was also observed that asthmatic patients admitted to hospital with influenza infection were less likely to die or require admission to intensive care compared with non-asthmatics. Using an *in vivo* model of asthma and influenza infection we demonstrate that prior exposure to *Blomia tropicalis* extract (BTE) leads to an altered immune response to influenza infection, comprised of less severe weight loss and faster recovery following infection. This protection was associated with significant increases in T cell numbers in the lungs of BTE sensitised and infected mice, as well as increased IFN-γ production from these cells. In addition, elevated numbers of CD11b+ dendritic cells (DCs) were found in the lung draining lymph nodes following infection of BTE sensitised mice compared to infected PBS treated mice. These CD11b+ DCs appeared to be better at priming CD8 specific T cells both *in vivo* and *ex vivo*, a function not normally attributed to CD11b+ DCs. We propose that this alteration in cross-presentation and more efficient T cell priming seen in BTE sensitised mice, led to the earlier increase in T cells in the lungs and subsequently faster clearance of the virus and reduced influenza induced pathology. We believe this data provides a novel mechanism that explains why asthmatic patients may present with less severe disease when infected with influenza.

## Introduction

Influenza is a respiratory virus that circulates in humans causing seasonal epidemics and sporadic pandemics. Globally, influenza epidemics result in approximately 3 to 5 million cases of severe illness and 250,000 to 500,000 deaths annually [1].

Asthma is a chronic inflammatory disease of the airways which is characterised by increased pulmonary eosinophilia, elevated Th2 cells and mucus hyper secretion [2]. Based on recent estimates as many as 334 million people currently suffer from asthma worldwide and the number of people diagnosed with asthma is on the rise [3, 4]. There are many triggers that can lead to the exacerbation of asthma, one of the most common being respiratory viral infections [5]. It has now been clearly shown that rhinovirus (RV) and respiratory syncytial virus (RSV) are two of the major respiratory viruses that can lead to asthma exacerbations [6–8]. In addition, asthma has also been identified as a risk factor for influenza. Epidemiological studies of the 2009 pandemic H1N1 outbreak demonstrated that even though asthma was found to be the most common underlying condition associated with hospitalization during the outbreak [9], a higher proportion of asthmatic patients were found to survive compared with patients with other underlying conditions [10]. In a separate analysis by McKenna *et al*, the majority of patients hospitalised with asthma and without pneumonia, were found to be less likely to need mechanical ventilation or require admission to the intensive care unit [11]. In another study conducted in the UK, asthmatics admitted to hospital were half as likely as non-asthmatics to die or require intensive care support, despite presenting with greater respiratory compromise at the time of hospital admission and similar rates of pneumonia [12]. Several *in vivo* studies have now indicated that pre-existing asthma can provide a protective effect against influenza induced disease through the production of either TGF-β or insulin-like growth factor-1 molecules from the epithelium [13, 14]. However, the role of dendritic cells (DCs) and T cells in mediating this protective effect have not been investigated. Dendritic cells in the lung can be broadly divided into three categories, plasmacytoid DCs, CD11b+ DCs and CD103+ DCs [15]. Many studies have now shown that CD11b+ DCs are important for the induction of asthma [16, 17], whilst CD103+ DCs have been shown to be important in the priming of CD8 T cells during an influenza infection [18–21]. Whilst these DC subsets have been shown to be crucial in the development and maintenance of asthma [15, 22] and the induction of the immune response to influenza [23, 24] it is unknown what happens to these subsets during a comorbidity model of asthma and influenza. Our findings demonstrate that asthma can indeed protect mice *in vivo* from influenza induced disease. We believe this is partially mediated by CD11b+ DCs in the lung draining mediastinal lymph nodes (MLN) which are able to cross-present to CD8 T cells in allergen sensitised mice, leading to the faster appearance of CD8 T cells in the lungs, quicker clearance of the virus and a reduction in virus induced pathology.

## Materials and methods

### Mice

C57BL/6 mice (8–10 weeks old) were purchased from National University of Singapore CARE. Mice were age and sex-matched for each experiment. Groups of five mice per cage were maintained under pathogen-free conditions and were transferred to the ABSL2 facility for experiments involving infection with influenza. Mice were randomly assigned to cages and each cage randomly assigned a condition as either a control or experimental group. The total number of mice used ranged from 10–20 depending on the experiment. All mice were allowed to acclimatise for 3–4 days prior to the start of the study. Mice were housed in individually ventilated cages and given access to food and water *ad libitum*. Prior to and during the experiments animals were monitored twice daily for health status. Mice were euthanized by flooding the chamber with 100% carbon dioxide gas at a flow rate 20–30% of chamber volume per minute. Flow rate was increased once the animals had lost consciousness. No adverse events were observed. All experiments were performed in strict accordance with the guidelines of the National Advisory Committee for Laboratory Animal Research (NACLAR), Singapore. The Institutional Animal Care and Use Committee (IACUC) of the National University of Singapore approved the protocols (Protocol numbers: 087/10 and 015/12). A completed ARRIVE guidelines checklist is included (S1 Fig).

### Allergen preparation

Ten grams of frozen *Blomia tropicalis* extract (Siriraj Dust Mite Centre for Services and Research, Thailand) was extracted overnight with slow stirring at 4°C in PBS (pH 7.4). The extract was then centrifuged at 13,000xg for 30 min at 4°C, and the supernatant was filtered through a 0.22-μm filter and stored at −80°C. The extract was assayed for endotoxin levels using the QCL-1000 kit (Hyglos GmbH, Bavaria, Germany) according to manufactures instructions and was less than 20 EU/mg of protein.

### Induction of allergic airways disease

C57BL/6 mice were anesthetised with a 3% isoflurane oxygen mixture and exposed to 0.5μg (20μl of a 25μg/ml protein weight solution in PBS) of *Blomia tropicalis* extract (BTE) or 20 μl of PBS intranasally (i.n) three times a week for two weeks.

### Virus propagation and infection of mice

Influenza virus strain A/PR/8/34 (H1N1) (VR-95) was purchased from American Type Culture Collection. Recombinant influenza A/PR/8/34 containing the chicken OVA epitope SIINFEKL (PR/8-OT-1) was a gift from Dr. Paul Thomas (St. Jude Children’s Research Hospital, Memphis, Tennessee, USA). Influenza viruses A/PR/8/34 (H1N1) and PR/8-OT-1 virus (with the SIINFEKL epitope) were grown in 10-day old embryonated chicken eggs as described previously [18]. Influenza virus was quantified by making serial ten-fold dilutions of virus that were allowed to adsorb onto confluent monolayers of Madin Darby Canine Kidney (MDCK) cells (ATCC CCL-34, ATCC, USA) on a 24-well plate for 1 hour at 37°C. The supernatant was then removed and replaced with 1% agarose supplemented with serum free Dulbecco’s Modified Eagle Medium (DMEM) (Invitrogen, Life Technologies, Singapore) and 2μg/ml TPCK (L(tosylamido-2-phenyl) ethyl chloromethyl ketone) treated Trypsin (Pierce, Research instruments, Singapore). Plates were incubated for 3 days at 37°C in 5% CO_2_. Agarose overlays were then removed and the plaques were visualized and enumerated after the addition of crystal violet stain. C57BL/6 mice were infected i.n. with 20μl of 10 PFU PR8 influenza virus or 100–500 PFU PR8-OVA virus, whilst under light anaesthesia with a 3% isoflurane oxygen mixture. Following infection weight change was monitored daily. Mice were euthanized prior to the endpoint if they lost more than 25% of their original weight.

### Isolation of cells from the bronchoalveolar lavage (BAL), lungs and mediastinal lymph nodes

Mice were sacrificed using carbon-dioxide asphyxiation. Bronchoalveolar lavage (BAL) was performed using three aliquots of 0.4ml PBS into the trachea of cannulated mice. BAL fluid was centrifuged (200xg, 5 min at 4°C) and supernatants were stored at -80°C for cytokine analysis. Cells were resuspended in 0.2ml red blood cell (RBC) lysis buffer (0.15M ammonium chloride, 1mM potassium hydrogen carbonate, 0.1mM disodium EDTA and 800ml of distilled water, pH 7.2) for 3 min at room temperature. 0.8ml of PBS containing 2% fetal calf serum and 5mM EDTA (flow buffer) was added and cells were centrifuged and resuspended in 0.5ml flow buffer before staining with the appropriate antibodies. To isolate cells from the lungs, lungs were first excised and chopped into smaller pieces and digested in 0.5mg/ml Liberase CI (Roche Diagnostics, Singapore) for 40 min at 37°C, before physical disruption into single cell suspension by filtration through a 70μm cell strainer (Fischer Scientific, Singapore). Single cell suspensions were treated with 3ml of RBC lysis buffer at room temperature for 5 min, topped up with 3ml of flow buffer and centrifuged (600xg, 5 min at 4°C). Cells were resuspended in 0.5ml or 1ml of complete media or flow buffer and filtered through a 70μm cell strainer.

Mediastinal lymph nodes (MLN) were excised and a single cell suspension obtained by physical disruption and filtration through a 70μm cell strainer. Cells were washed in flow buffer and treated with RBC lysis buffer for 2 min at room temperature. Cells were topped up with flow buffer, centrifuged (600xg, 5 min at 4°C) and resuspended in 1ml of flow buffer.

### Determination of viral titer in the lung

Viral titers in the lungs were determined by quantitative reverse transcription PCR (qRT-PCR) on viral mRNA as described previously [25]. Briefly, total RNA was isolated from the lung using an RNeasy kit (Qiagen, Singapore) according to the manufacturer’s instruction. cDNA was synthesized using the High capacity cDNA reverse transcription kit (Applied Biosystems, Singapore). Real-time qPCR was performed on an ABI 7500 real-time PCR system (Applied Biosystems, Singapore) using the GoTaq qPCR master mix containing BRYT green (Promega, Singapore). Primers used for qPCR were as follows: Influenza M-protein forward primer 5’-GGACTGCAGCGTTAGACGCTT-3’; Influenza M-protein reverse primer 5’- CATCCTGTTGTATATGAGGCCCAT-3’; beta-actin forward primer 5’- AGAGGGAAATCGTGCGTGAC-3’; beta-actin reverse primer 5’- CAATAGTGATGACCT GGCCGT-3’.

### Flow cytometry and cell sorting

Prepared cells were washed with PBS twice and stained with the LIVE/DEAD fixable dead cell stain kit (Invitrogen, Singapore) for 20 min at 4°C in the dark. Cells were then washed in PBS, resuspended in flow buffer and blocked with Fc block (anti- CD16/32; BD Bioscience, Singapore) for 20 min at 4°C in the dark. Cells were washed in flow buffer and stained for surface markers for 30 min at 4°C in the dark. Following staining cells were washed twice in flow buffer and fixed using 1% paraformaldehyde. For intracellular staining, prepared cells were stimulated for 4 hours with phorbol 12-myristate 13-acetate (PMA) (50 mg/ml) and ionomycin (500 ng/ml) (Sigma, Singapore) in the presence of 5μg/ml monensin and brefeldin A (BD Bioscience, Singapore) at 37°C. Cells were then surfaced stained as described above and fixed and permeabilized using the eBioscience fixation/permeabilization buffer (eBioscience, Singapore) overnight. The next day cells were permeabilized using the permeabilization buffer (eBioscience, Singapore) and stained for intracellular cytokines for 30 min at 4°C in the dark. Cells were then washed twice in permeabilization buffer and resuspended in flow buffer. The following antibodies were purchased from BD BioScience, Singapore. Anti-CD8 (clone 53–6.7), anti-IFN-γ (clone XMG1.2), anti-Siglec-F (clone E50-2440), anti-Ly6G (clone 1A8) and anti-NK1.1 (clone PK136). The following antibodies were purchased from eBioscience, Singapore. Anti-CD3 (clone 145-2C11), anti-CD4 (clone RM4-5), anti-CD11c (clone N418), anti-MHC Class II I-A/I-E (clone M5/114.15.2), anti-CD11b (clone M1/70), anti-CD103 (clone 2E7), anti-F4/80 (clone BM8) and anti-19 (clone eBio 1D3). Pentamer positive cells were stained for after FC blocking for 15 min at 4°C, prior to adding the rest of the antibodies. R-PE labelled Pro5 MHC Pentamer for H-2Db/ASNENMETM binding to NP_366_ was purchased from ProImmune Ltd (Oxford, UK). Cells were run on a LSR Fortessa or X-20 flow cytometer (BD Bioscience, Singapore). Data were analysed using the Flowjo analysis program, version 10.0.8 (Ashland, Oregon, USA). The gating strategy used to identify eosinophils, neutrophils, macrophages and DCs is shown by the flow plots in S2 Fig. To analyse T cell, B cell and NK cell populations cells were first gated on live cells, followed by doublet cell exclusion. A FSC vs SSC plot was used to identify the lymphocyte gate from which gates for T cells, B cell and NK cells were established (flow plots not shown). Total live cell counts were obtained by trypan blue exclusion and the total number of each cell type was calculated by the following formula using the counts obtained from the Flowjo analysis ((Total live cell count/Gated live cell count) x Count from the gated population of interest). For cell sorting experiments, cells were blocked and stained with antibodies as described above, but without the initial live/dead stain. Cells were sorted using a Sy3200 cell sorter (Sony Biotechnology, San Jose, California, USA). To isolate CD103+ and CD11b+ DCs single live cells were first gated on (Plots A and B in S3 Fig). A dump channel was then used to remove CD3+, CD4+, CD8+ cells, NK cells, macrophages and B cells. Cells that were negative for these markers were gated around (Plot C in S3 Fig) and MHC class II+ high and CD11c+ high cells identified (Plot D in S3 Fig). CD103+ and CD11b+ DCs were then identified and isolated (Plot E in S3 Fig). The purity for both DC populations were between 97–98%.

### Albumin quantification in the BAL

Albumin in BAL supernatants were quantified by ELISA using the Mouse Albumin ELISA Quantitation Set from Bethyl Laboratories, Inc (Montgomery, Texas, USA) according to manufactures instructions. Briefly, Immunosorb ELISA plates (Nunc, Singapore) were coated with purified antibody for 60 min at room temperature. Wells were washed five times and blocking solution added for 30 min at room temperature. Plates were washed five times and samples or standards were added for 60 min at room temperature. Plates were washed five times and horseradish peroxidase detection antibody added for 60 min at room temperature. Plates were washed five times and tetramethylbenzidine was added. Plates were incubated at room temperature in the dark. The enzymatic colour reaction was stopped using 1M H_2_SO_4_ and optical densities read at 450nm. The concentration of albumin was determined from the standard curve.

### OT-1 CD8 T cell isolation and adoptive transfer

Spleen and lymph nodes were obtained from naïve female OT-1 C57BL/6 mice. Lymph nodes were processed as previously described above. To obtain a single cell suspension, spleens were physically disrupted and filtered through a 70μm cell strainer, topped up with flow buffer and centrifuged (600xg, 5 min at 4°C). Red bloods cells were lysed with ACK lysis buffer and CD8+ T cells were isolated using the EasySep Mouse CD8 T cell Isolation Kit according to manufactures instructions (STEMCELL Technologies, Singapore). Isolated CD8+ T cells were labelled with 5μM CellTrace Violet dye (Thermo Fisher Scientific) according to manufacturer’s instructions resuspended in Hank’s Balanced Salt Solution at 2x10^7^/ml. For adoptive transfer 100μl of the cell suspension was injected via the retro-orbital route into mice anesthetised with isoflurane.

### OT-1 CD8 T cell proliferation assays

OT-1 CD8+ T cells were isolated and labelled with CellTrace Violet as described above. CD11b+ and CD103+ DC’s were isolated from the MLN of PBS treated or BTE sensitised mice that were infected with PR8-OVA. Single cell suspensions were obtained by passing the MLNs through a 75μm nylon mesh filter. Cells were blocked with Fc block (anti- CD16/32; BD Bioscience, Singapore) for 20 min at 4°C in the dark and then stained with the appropriate antibodies for 30 mins at 4°C in the dark. DC populations were isolated by fluorescence activated cell sorting using the gating strategy displayed in S3 Fig as described above. Isolated CD11b+ DC’s and CD103+ DCs were co-cultured with labelled OT-1 CD8 T cells in a round bottom 96-well plate at a ratio of 1:10 (DC: T cell) for 3 days at 37°C, 5% CO_2_. On the third day cells were harvested and stained for CD8 T cells and CellTrace violet dilution was measured by flow cytometry.

### Histopathology

For haematoxylin and eosin (H&E) and periodic acid-Schiff (PAS) staining the chest cavity was opened and the lungs exposed. The aortic artery cut to release blood flow. Lungs were then inflated with 3x10ml of PBS through the heart or until the lungs turned white. A cannula was inserted into the trachea and 1ml of 4% paraformaldehyde (PFA) injected to fix the lungs. Lungs were then immersed in 4% PFA. Paraffin-embedded sections (4μm) were cut and stained with H&E. A semi-quantitative scoring system was used to grade the size of lung infiltrates as previously described [26]. Briefly, a score of 5 signified a large (> 3 cells deep) widespread inflammatory infiltrate around the majority of vessels and bronchioles, and a score of 1 represented a small (≤ 2 cells deep) number of inflammatory foci. For goblet cell hyperplasia analysis, sections were stained with PAS and a semi-quantitative scoring system was used where positively stained cells in the airway epithelium were measured (0 = < 5% goblet cells; 1 = 5–25%; 2 = 25–50%; 3 = 50–75%; 4 = > 75%). The sum of the airway scores from each lung was divided by the number of airways examined (20–40 per mouse), and expressed as mucus score in arbitrary units.

### Statistical analysis

Statistical analysis was performed using GraphPad Prism software (La Jolla, California, USA). Comparisons across groups were made using 1-way or 2-way ANOVA and paired comparisons were determined using the non-parametric Mann-Whitney test. Data are expressed as means ± SEM. * *p*< 0.05; ** *p*< 0.01 and *** *p*<0.001 are indicated in Figures.

## Results

### Induction of asthmatic phenotype following low dose exposure to BTE

**Fig 1:**
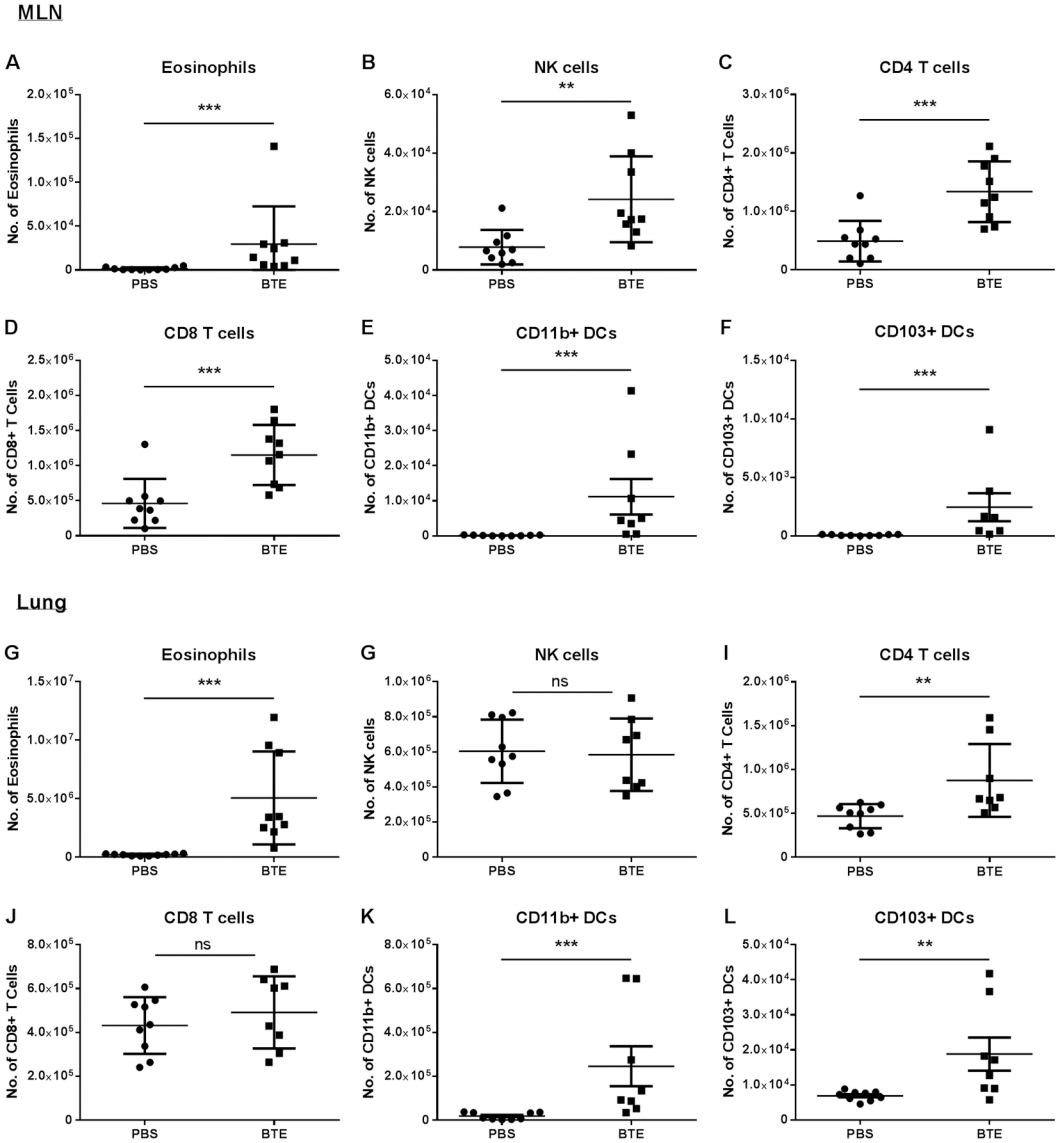
Low dose BTE exposure leads to the induction of the asthmatic phenotype. Mice were sensitized with either PBS or 0.5μg of BTE 3 times a week, for 2 weeks. 24 hr after the last sensitization mice were culled and cellular infiltrate into the MLN and lungs were measured. The total number of (A) eosinophils, (B) NK cells, (C) CD4 T cells, (D) CD8 T cells, (E) CD11b+ DCs and (F) CD103+ DCs in the MLN are shown. The total number of (G) eosinophils, (H) NK cells, (I) CD4 T cells, (J) CD8 T cells, (K) CD11b+ DCs and (L) CD103+ DCs in the lungs are shown. Graphs represent mean ± SEM representing pooled date from two independent experiments with n≥5 mice in each group. Significant differences between PBS and BTE treated mice are shown as *p<0.05; **p<0.01 and ***p<0.001.

C57BL/6 mice were exposed to either PBS or a low dose of *B*. *tropicalis* extract (BTE) (0.5μg) intranasally (i.n.) three times a week for two weeks, culled twenty-four hours after the last exposure and the mediastinal lymph nodes (MLN) and lungs assessed for inflammatory cell infiltrate. Following sensitisation with BTE there was a significant increase in the number of eosinophils (Fig 1A), NK cells (Fig 1B), CD4 T cells (Fig 1C) and CD8 T cells (Fig 1D) in the MLN. No difference was found in the number of neutrophils (data not shown). In addition, sensitisation led to a significant increase in two important DC subsets in the MLN, CD11c^hi^ MHC class II^hi^ CD11b+ (CD11b+) (Fig 1E) and CD11c^hi^ MHC class II^hi^ CD103+ (CD103+) (Fig 1F). Whilst both DC populations were found to be increased, there were significantly more CD11b DCs compared to CD103+ DCs. A similar pattern was observed in the lungs of BTE sensitised mice compared to PBS treated mice for eosinophils, CD4 T cells, CD11b+ DCs and CD103+ DCs (Fig 1G, 1I, 1K and 1L). However, unlike the MLN, there was no significant difference in the number of NK cells or CD8 T cells in the lungs of sensitised mice (Fig 1H and 1J).

### Prior sensitization to BTE reduced influenza induced weight loss and led to faster clearance of the virus with less sustained lung damage

**Fig 2:**
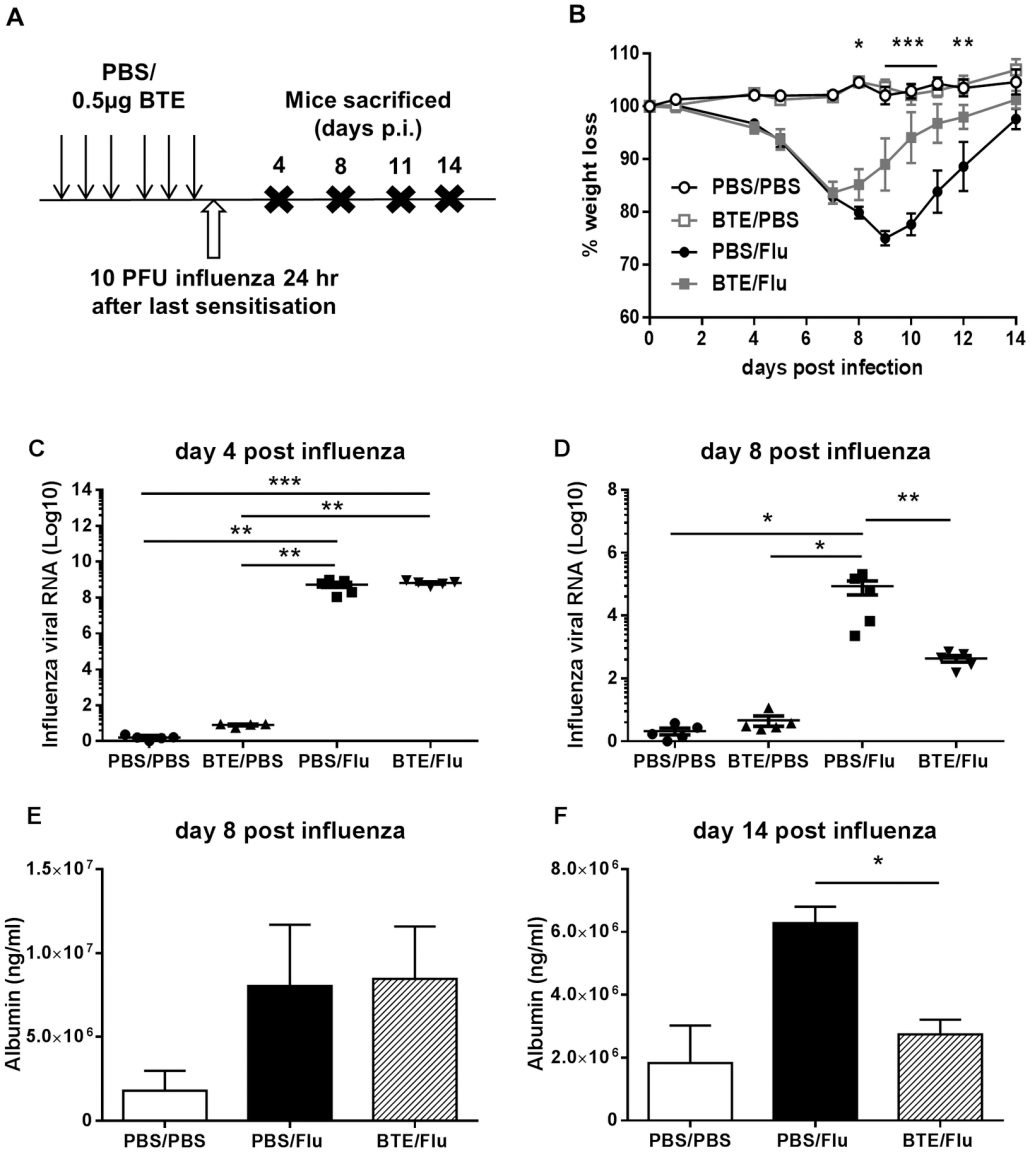
Prior sensitization to BTE reduces influenza induced weight loss. (A) Protocol for sensitization with BTE and influenza infection. Mice were sensitized with either PBS or 0.5μg of BTE 3 times a week, for 2 weeks. 24 hr after the last sensitization mice were infected with 10 PFU of influenza virus. Mice were culled at days 4, 8, 11 and 14 p.i. (B) Weight loss following influenza virus infection. Levels of mRNA for the matrix protein are shown at (C) day 4 and (D) day 8 p.i. Albumin leakage into the BAL was assessed by ELISA at (E) day 8 and (F) day 14 p.i. Symbols and bars represent individual animals or the mean ± SEM. Graphs represent data from one of two independent experiments with n≥5 mice in each group per time point. Significant differences between infected and non-infected groups and between BTE/Flu and PBS/Flu treated mice are shown as *p<0.05; **p<0.01 and ***p<0.001.

Having established an asthmatic phenotype using low dose BTE sensitisation, mice were subsequently infected with 10 PFU of influenza (PR8) twenty-four hours after the last sensitisation and culled at various time points thereafter as shown in the schedule (Fig 2A). As a control group mice were treated with PBS instead of BTE and infected twenty-four hours after the last PBS dose. Weight loss was monitored every day for two weeks and those sensitised prior to influenza started to lose weight at the same time and rate as the infected PBS treated mice. However, infected BTE sensitized mice stopped losing weight earlier at day 7 post infection (p.i.) compared to infected PBS treated mice whose weight loss peaked at day 9 p.i. A faster recovery from weight loss was also seen in the infected BTE sensitised mice (Fig 2B). Having seen less weight loss and faster recovery in infected BTE sensitised mice, viral loads were measured on day 4 and day 8 p.i. Interestingly, viral loads on day 4 p.i. from both the infected BTE sensitised and PBS treated mice were similar and significantly higher than control PBS and BTE sensitised non-infected groups (Fig 2C). However, at day 8 p.i. there was a significant decrease in viral load detected in the lungs of infected BTE sensitised mice compared to infected PBS treated mice (Fig 2D). As an indicator of lung damage the level of albumin leakage into the BAL was also measured. At day 8 p.i. no difference was seen between infected PBS treated or BTE sensitised mice (Fig 2E). However, at day 14 p.i. there was a significant decrease in the amount of albumin that was detected in the BAL of infected BTE sensitised mice (Fig 2F), suggesting there was less ongoing epithelial lung damage in mice which were BTE sensitised and infected.

### Earlier appearance of acute inflammation and goblet cell hyperplasia in BTE sensitised mice

**Fig 3:**
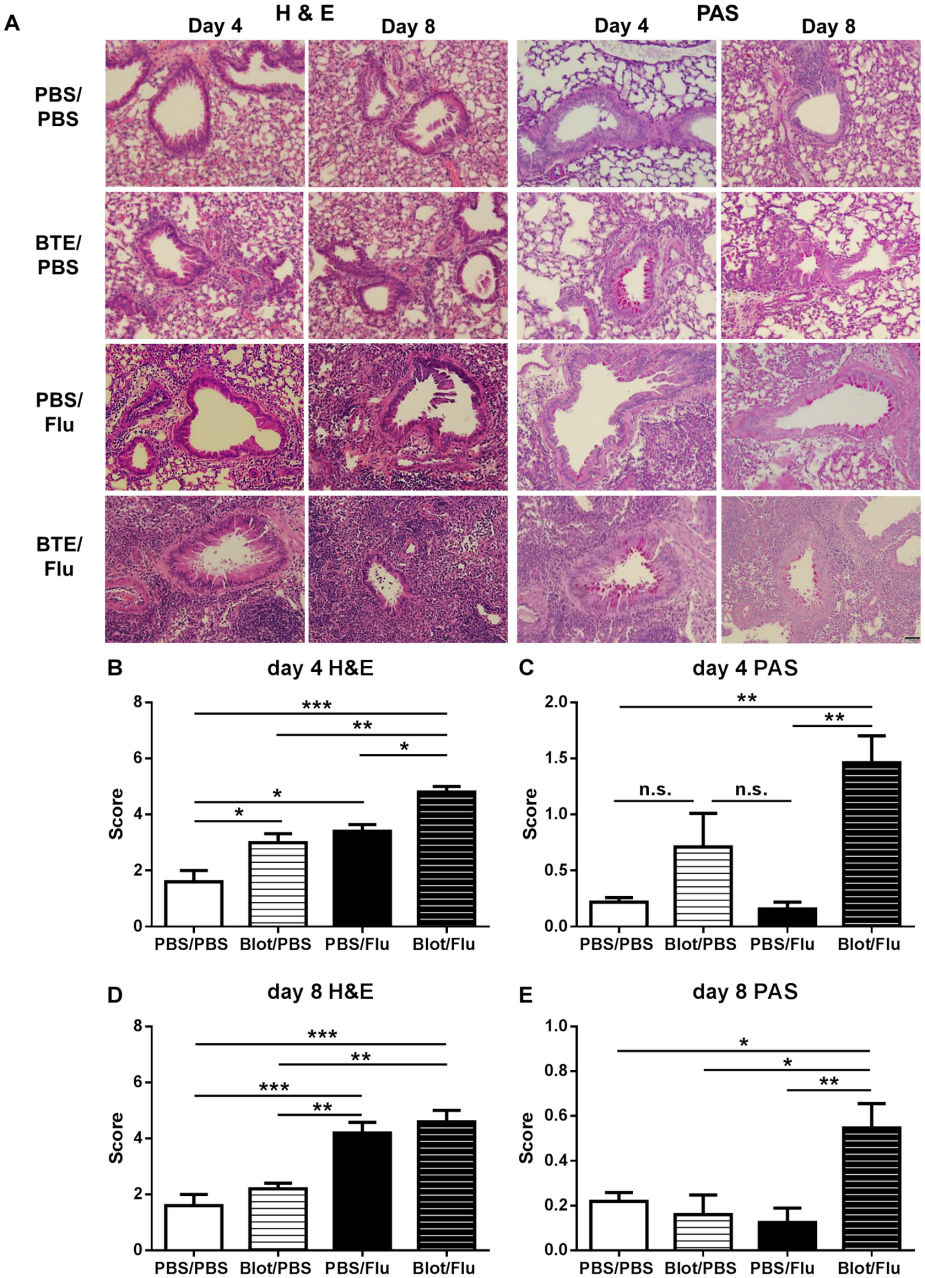
Earlier increase in lung inflammation and sustained mucus production in the lungs of infected BTE treated mice. Mice were sensitized and infected as described previously. Lungs were harvested on day 4 and 8 p.i., sectioned and stained with H&E and PAS. (A) Representative images of airways stained for H&E and PAS are shown for day 4 and day 8 p.i. Semi-quantitative scoring was performed for H&E stained sections at (B) day 4 and (D) day 8 p.i. and for PAS stained sections at (C) day 4 p.i. and (E) 8 p.i. Original magnification X40. Scale bar = 50μm. Graphs represent means ± SEM from one of two independent experiments with n≥5 mice in each group per time point. Significant differences between groups are shown as *p<0.05; **p<0.01 and ***p<0.001.

To assess the level of inflammation in the lungs of infected BTE sensitised mice, lung sections were obtained from non-infected and infected PBS treated or BTE sensitised mice at day 4 and 8 p.i. H&E staining showed there was a significant increase in inflammation at day 4 p.i. in the lungs of infected BTE sensitised mice compared to infected PBS treated mice and non-infected PBS treated and BTE sensitised mice (Fig 3A and 3B). In addition to increased inflammation, there was also a significant increase in the number of mucus secreting goblet cells in the lungs of infected BTE sensitised mice compared to infected PBS treated mice or the non-infected groups (Fig 3A and 3C). At day 8 p.i., the inflammation score of infected PBS treated or BTE sensitised mice was comparable, with a high inflammation score seen in both groups (Fig 3A and 3D). However, unlike the inflammation level, mucus producing goblet cells were only visible in the lungs of infected BTE sensitised mice (Fig 3A and 3E).

### BTE exposure prior to influenza infection results in increased airway NK cells and T cells and the faster expansion of influenza-specific CD8 T cells in the lungs

**Fig 4:**
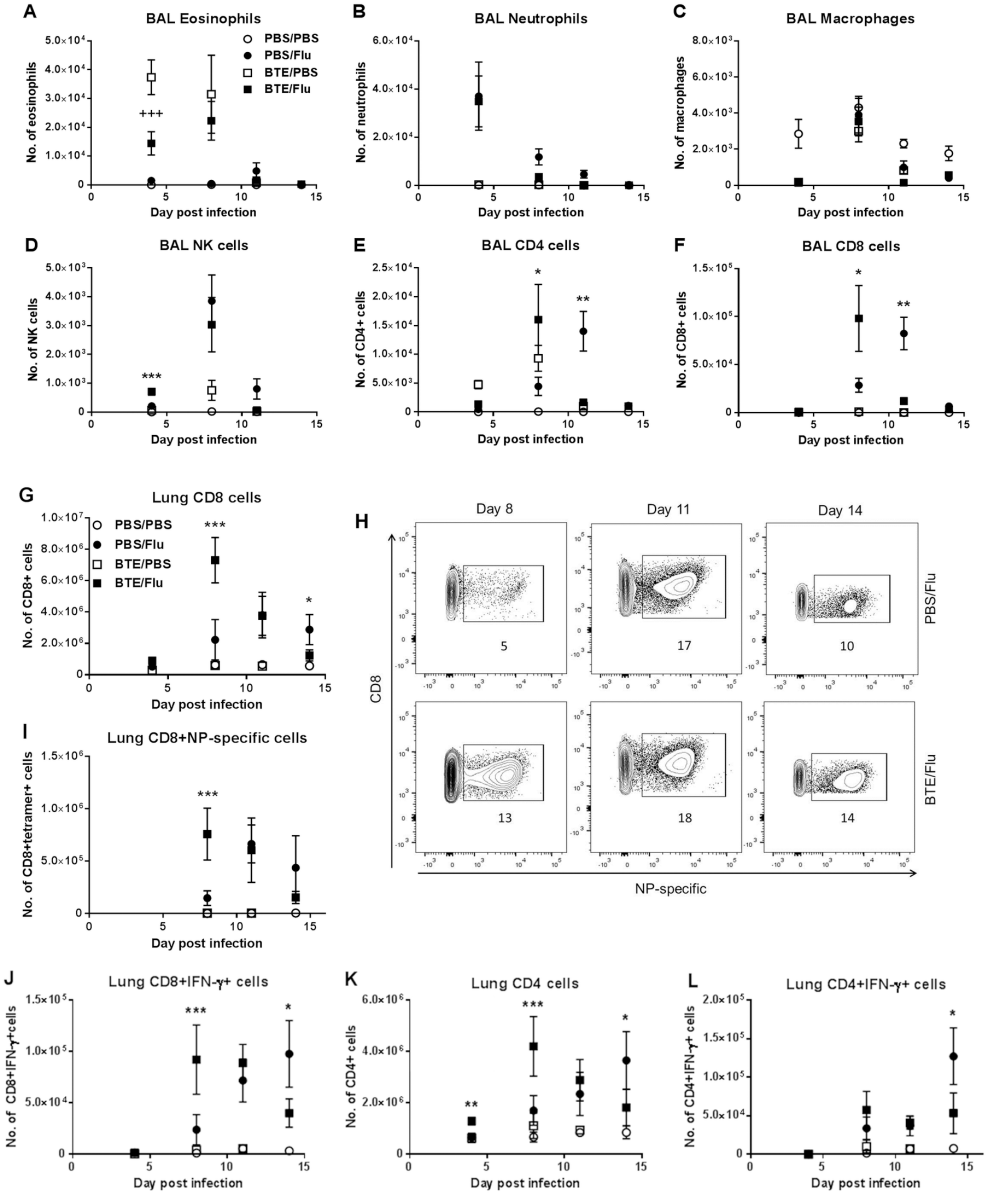
Prior sensitization to influenza leads to enhanced NK and T cell recruitment to the airways and lungs. The total number of (A) eosinophils, (B) neutrophils, (C) macrophages, (D) NK cells, (E) CD4 and (F) CD8 T cells in the BAL is shown at days 4, 8, 11 and 14 p.i. Lung cells were stimulated ex vivo for 4 hr with PMA and ionomycin and surface and intracellular cytokine staining was performed. Total number of (G) CD8 T cells, (I) CD8+NP-specific cells, (J) CD8+IFN-γ+ cells, (K) CD4 T cells and (L) CD4+IFN-γ+ cells in the lungs are shown at days 4, 8, 11 and 14 p.i. (H) Representative flow plots indicating the frequencies of NP-specific CD8 T cells are shown at days 8, 11 and 14 p.i. Graphs represent means ± SEM of pooled data from at least two independent experiments with n≥5 mice in each group per time point. Significant differences between BTE/Flu and BTE/PBS treated mice are shown as +++p<0.001 and between BTE/Flu and PBS/Flu treated mice are shown as *p<0.05; **p<0.01 and ***p<0.001.

To understand which cells were involved in causing the earlier increase in inflammation and possibly in turn, the protection of sensitised mice from subsequent influenza pathology, innate and adaptive infiltrating cells in the airway were analysed at various time points after infection. As expected the majority of changes in the granulocyte population occurred around day 4 p.i. Airway eosinophil levels were highest in the non-infected BTE sensitised mice and were significantly higher than in the infected BTE sensitised mice (Fig 4A), suggesting that infection had inhibited the Th2 inflammatory response. The number of neutrophils and macrophages were comparable in both the BTE sensitised and PBS infected mice (Fig 4B and 4C). Furthermore, there was a small, but statistically significant increase in the number of NK cells in the airway of infected BTE sensitised mice compared to infected PBS treated mice at day 4 p.i., which was no longer seen by day 8 p.i. (Fig 4D). Finally, there was a significant increase in the number of CD4 and CD8 T cells detected in the airways at day 8 p.i. in infected BTE sensitised mice, whereas the number of T cells in infected PBS treated mice peaked at day 11 p.i. (Fig 4E and 4F).

Having seen a greater increase in T cell numbers in the airways (BAL), T cells within the lungs were analysed in more detail at various time points after infection. As seen in the airway, the number of CD8 T cells within the lungs of infected BTE sensitised mice were very low on day 4 p.i., but rose rapidly peaking on day 8 p.i., much earlier and significantly higher than the peak seen in infected PBS mice which occurred at day 11 p.i. (Fig 4G). In addition, an increase in the frequency and number of influenza NP-specific CD8 T cells (Fig 4H and 4I) and CD8 T cells producing IFN-γ (Fig 4J) was also seen at day 8 p.i. in infected BTE sensitised compared to infected PBS treated mice. This earlier increase in CD8 T cells in infected BTE sensitised mice was accompanied by a quicker decline in CD8 T cells in the lungs and by day 14 p.i. the number of CD8 T cells was significantly lower compared to infected PBS treated mice (Fig 4G). This was also reflected in the number of CD8 T cells producing IFN-γ at day 14 p.i. (Fig 4J). Concomitant with the early increase in CD8 T cells a similar pattern was observed for CD4 T cells, with significantly higher numbers of CD4 T cells (Fig 4K) seen at day 4 and day 8 p.i. in infected BTE sensitised mice compared to infected PBS treated mice. A similar number of CD4 T cells producing IFN-γ were found in both infected BTE sensitised and PBS treated mice between day 4 and 11 p.i. However, at day 14 p.i. a significant increase was seen in infected PBS treated mice compared to infected BTE sensitised mice (Fig 4L).

### Augmented innate immune and adaptive immune response in the MLN of asthmatic influenza infected mice

**Fig 5:**
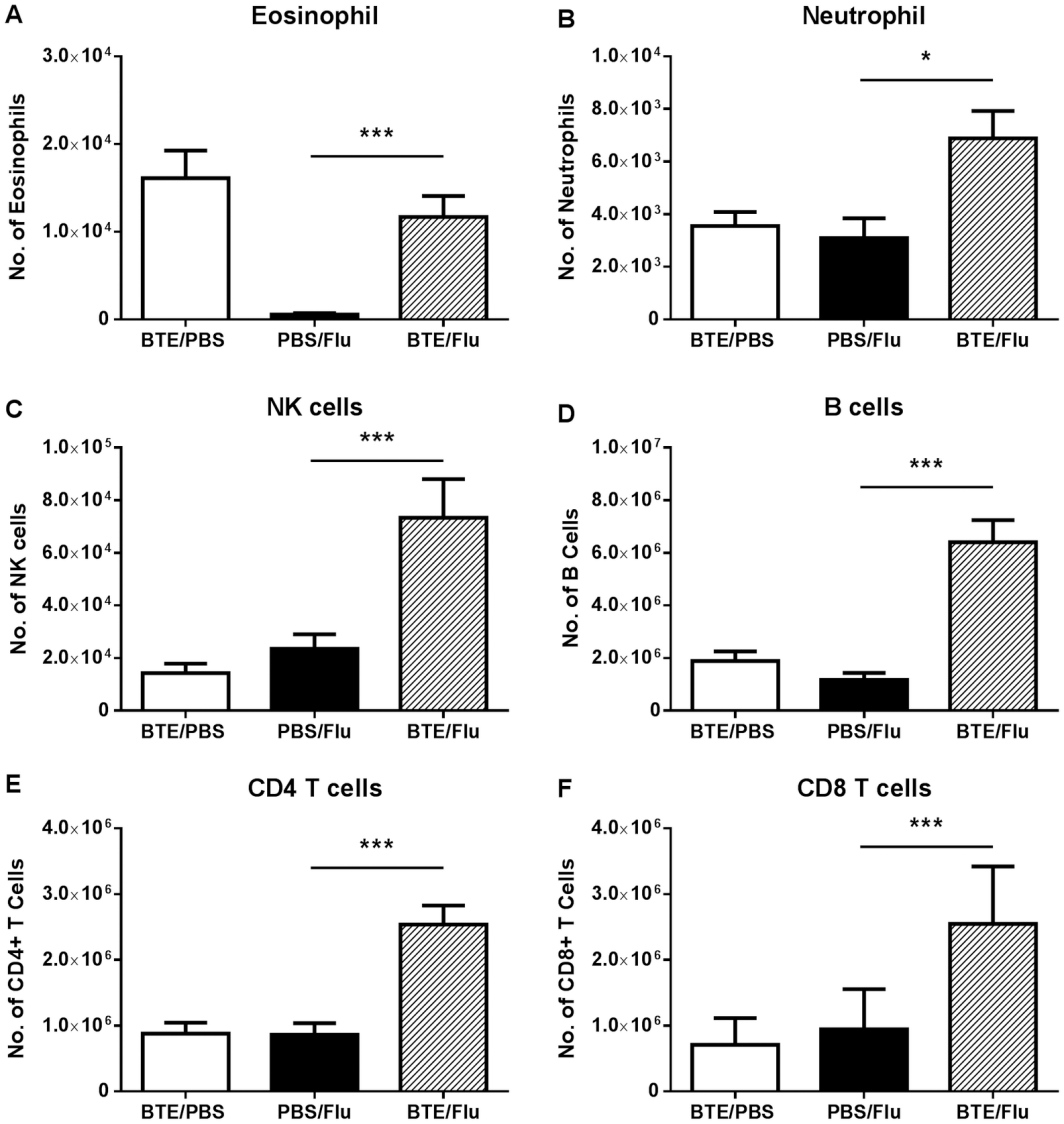
Augmented innate immune and adaptive immune response in the MLN of asthmatic influenza infected mice. MLN from PBS and BTE exposed and infected mice were analysed at day 3 p.i. The total number of (A) eosinophils, (B) neutrophils, (C) NK cells, (D) B cells, (E) CD4 T cells and (F) CD8 T cells are shown. Graphs represent means ± SEM of pooled data from at least two independent experiments with n≥5 mice in each group. Significant differences between BTE/Flu and PBS/Flu treated mice are shown as *p<0.05 and ***p<0.001.

As the immune response in infected BTE sensitised mice appeared to begin earlier than that of infected PBS treated mice we analysed the initial phase of the influenza infection, in particular which cells were present in the MLN during the early phase of the immune response to the virus. BTE sensitised or PBS treated mice were infected with influenza and at day 3 p.i. their MLN analysed for innate and adaptive immune cells. BTE sensitised and infected mice had a significant increase in a number of innate immune cells, including eosinophils, neutrophils and NK cells, compared to PBS infected mice (Fig 5A–5C). There was also a significant increase in the number of eosinophils in the non-infected BTE group, indicating there was a sustained ongoing Th2 immune response, even three days after the last sensitisation (Fig 5A). In addition, these mice also had significantly higher numbers of B cells (Fig 5D), CD4 (Fig 5E) and CD8 T cells (Fig 5F).

### Altered DC recruitment to the MLN in asthmatic mice infected with influenza

**Fig 6:**
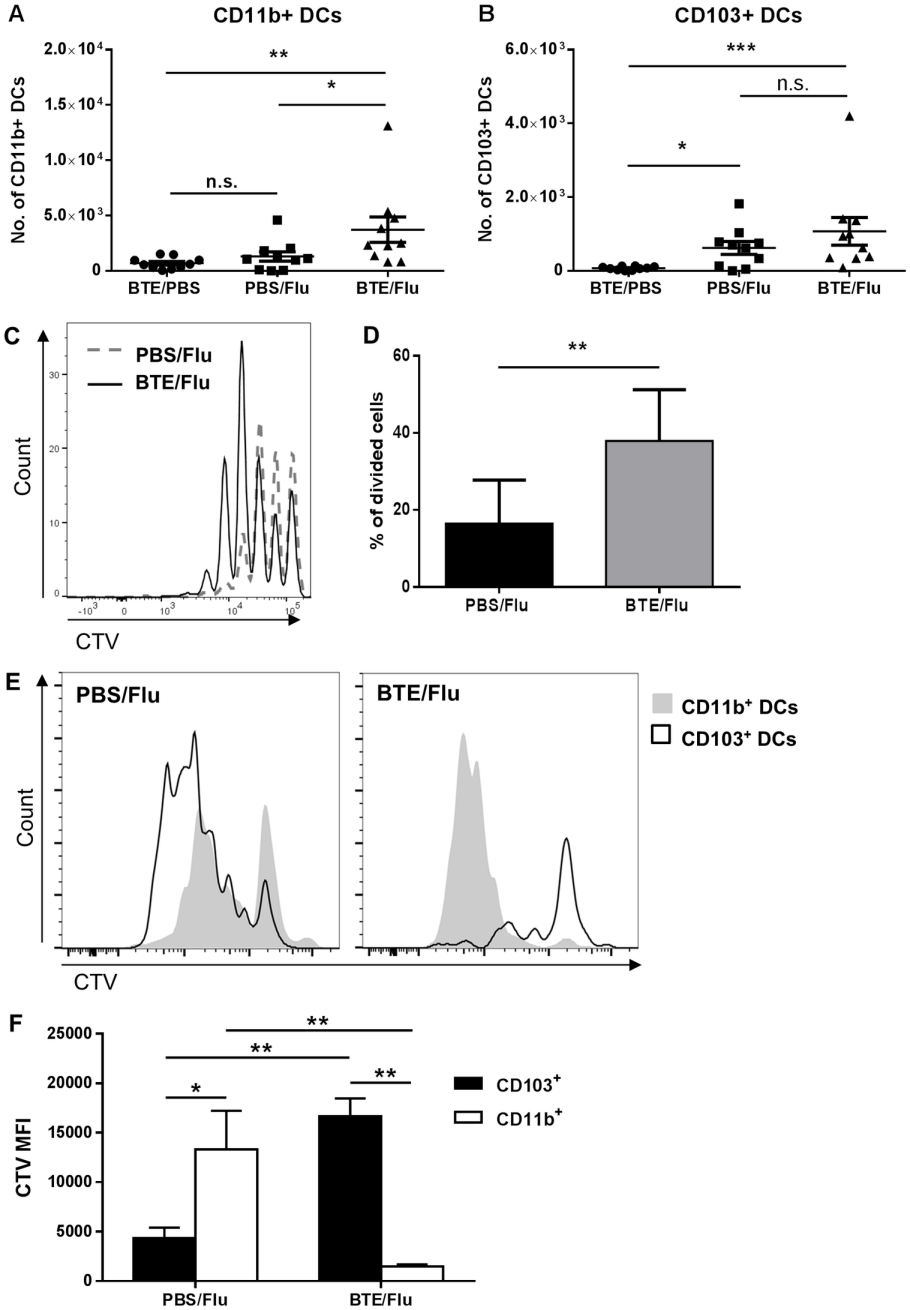
Altered recruitment of DCs and earlier T cell proliferation in the MLN of asthmatic mice infected with influenza. Mice were sensitized with either PBS or BTE 3 times a week, for 2 weeks. 24 hr after the last sensitization mice were infected with either influenza PR8 or influenza PR8-OVA virus. Mice were culled at day 3 p.i. and the total numbers of (A) CD11b+ and (B) CD103+ DCs were enumerated in the MLN. (C) and (D) Representative histogram and graph showing proliferation of adoptively transferred OT-1 CD8 T cells labelled with CTV, measured 3 days after infection with PR8-OVA. (E) and (F) Representative histograms and graph comparing CTV MFI of labelled OT-1 CD8 T cells cultured with sorted CD103+ DCs or CD11b+ DCs from PBS or BTE infected mice at 3 p.i. Symbols and bars represent individual animals with the mean ± SEM. Graphs represent pooled data from two independent experiments with n≥5 mice in each group. Significant differences are shown as *p<0.05; **p<0.01 and ***p<0.001, n.s; not significant.

To investigate the reason for the earlier increase in T cells seen in the MLN and the lungs of infected BTE sensitised mice, the number of CD11b+ and CD103+ DCs were analysed in the MLN at day 3 p.i. Mice sensitised with BTE and infected with influenza showed a significant increase in the number of CD11b+ DCs compared to infected PBS treated mice and non-infected BTE sensitised mice (Fig 6A). There was no difference in the number of CD103+ DCs detected in either infected BTE sensitised or infected PBS treated mice. However, there was a significant increase in the number of CD103+ DCs in both infected groups compared to non-infected BTE sensitised mice (Fig 6B), indicating that influenza infection was responsible for the trafficking of CD103+ DCs to the MLN, as shown previously [18].

To study the T cell response to influenza, CellTrace Violet (CTV) labelled OT-1 CD8 T cells were adoptively transferred into either PBS treated or BTE sensitised mice that were then infected with the recombinant influenza virus PR8-OVA. This virus contains the OVA epitope SIINFEKL that is specifically recognised by OT-1 CD8 T cells. Mice were culled at day 3 p.i. and their MLN analysed for the proliferation of OT-1 CD8 T cells. Sensitisation with BTE prior to infection led to a significant increase in the proliferation of OT-1 CD8 T cells in the MLN compared to infected PBS treated mice, as seen by the increased number of proliferation cycles and by the increased percentage of divided cells, 16% in infected PBS treated mice versus 37% in infected BTE sensitised mice (Fig 6C and 6D). There were no CTV labelled OT-1 CD8 T cells detected in the lungs of either PBS treated or BTE sensitised non-infected mice at this time (data not shown).

To further identify if the enhanced proliferation was due to augmented DC presentation by CD103+ DCs from BTE sensitised mice, or if in these mice the CD11b+ DCs were now able to cross-present to CD8 T cells, both DC populations were sorted by flow cytometry from the MLN of PBS treated or BTE sensitised mice that were infected with PR8-OVA, at day 3 p.i. Sorted DCs were cultured with naïve CTV labelled OT-1 CD8 T cells for three days, after which their proliferation was analysed. CD103+ DCs from infected PBS treated mice induced significant proliferation of OT-1 CD8 T cells, as seen by their CTV dilution, compared to CD11b+ DCs from the same mice. In contrast, in BTE sensitised mice significant CD8 T cell proliferation was only seen in the presence of CD11b+ DCs, and not CD103+ DCs, indicating that CD11b+ DCs were the main DC population presenting antigen to OT-1 CD8 T cells and responsible for the enhanced T cell proliferation seen earlier (Fig 6E and 6F).

## Discussion

It has long been thought that individuals with asthma may be more susceptible to respiratory viral infections, and whilst a link between the two has been shown for RSV and RV [6–8], epidemiological studies involving influenza have yielded conflicting results [9–11].

The findings presented here show that prior exposure to low dose allergen can promote a beneficial immune response to subsequent influenza viral infection. Infected allergen exposed mice had augmented innate and adaptive immune responses, seen in the lung draining lymph nodes (MLN), lungs and airways, which led to the earlier recruitment of CD4 and CD8 T cells, as well as influenza specific CD8 T cells into the lungs. The result of this earlier inflammation of the lungs was faster clearance of the virus, reduced viral induced weight loss and reduced lung damage.

Others have also addressed how the immune response to influenza infection can be altered in an allergic setting. Using an *Aspergillus fumigatus* model of asthma Samarasinghe *et al* showed that infection during the acute phase of asthma leads to protection from influenza virus induced weight loss and disease. However, infection during chronic asthma led to the loss of this protective effect and mice were found to develop lung pathology comparable to that of infected PBS treated mice. They believed the protective effect seen in acute asthmatic mice was due to the maintenance of an intact epithelium, reduced interferon responses and increased production of insulin-like growth factor-1 [14]. In a more recent study Furuya *et al* concluded that influenza induced pathology can be prevented by the transient increase in production of TGF-β during allergic asthma. They show that asthmatic mice infected with influenza displayed a reduced cytokine storm and cytotoxicity in the airways and that by deleting TGF-β receptor II they abrogated the resistance of asthmatic mice to influenza [13]. From the data presented here we suggest that CD11b+ DCs play an important role in the protective effects seen in BTE sensitised mice infected with influenza. We show that low dose sensitisation with BTE led to the increase in CD11b+ and CD103+ DCs in the MLN and the lungs. Although both DC populations were found to increase, the magnitude of the increase in CD11b+ DCs was significantly larger. This is perhaps not surprising as CD11b+ DCs have been shown to rapidly translocate to the MLN following allergen exposure. It has also been demonstrated that in the presence of low allergen dose, conventional CD11b+ DCs and not monocyte-derived DCs are the main DCs to migrate to the MLN and present to CD4 T cells priming for a Th2 immune response [16, 17]. In contrast, during a primary influenza infection both CD11b+ and CD103+ DCs have been shown to capture viral antigen and transport it to the lung draining lymph nodes. However, in this situation only the CD103+ DCs present viral antigen to naïve CD8 T cells [18, 19, 27]. We propose that prior exposure to BTE, before influenza infection, leads to lung inflammation, activation and recruitment of DCs and the disruption of the airway epithelium, which exposes lung CD11b+ DCs normally located in the submucosa and lung parenchyma [28] to viral antigen, which they capture and then carry to lung draining lymph nodes where they cross-present to CD8 T cells. In support of this we have shown that influenza infection following sensitisation resulted in a significant increase in CD11b+ DCs in the MLN, compared to infected PBS treated mice. In contrast, numbers of CD103+ DCs in the MLN were found to be similar in both infected PBS and BTE treated mice, with both higher than in non-infected BTE treated mice. In addition, we also found that the *in vivo* proliferation of transferred naive OT-1 CD8 T cells within the MLN was greater in infected BTE sensitised mice compared to infected PBS treated mice, suggesting that there was a significant increase in antigen presentation to CD8 T cells in these mice. To identify which DC population was responsible for this increased proliferation we sorted CD103+ and CD11b+ DCs from infected PBS treated or BTE sensitised mice and co-cultured them with naïve OT-1 CD8 T cells. To our surprise, we found that in infected BTE sensitised mice the enhanced T cell proliferation came from T cells co-cultured with CD11b+ DCs and not CD103+ DCs. The idea that CD11b+ DCs are able to acquire and present influenza antigen is not new. Several groups have shown that CD11b+ DCs are more efficient in viral antigen uptake in the lung, compared to CD103+ DCs they migrate poorly to the lung draining lymph nodes during the early stages of the immune response to primary influenza infection [18, 20, 27, 29]. However, Ballesteros-Tato *et al* have demonstrated that during the peak of the T cell response to influenza infection (day 5–10 p.i.), lung CD11b+ DCs are able to capture viral antigen, process the antigen into the MHC class I pathway and migrate to the lung draining lymph nodes where they cross-present to naïve CD8 T cells [30]. Although both CD103+ and CD11b+ DCs can be infected by influenza, a number of groups have shown that the virus is only able to propagate in the CD103+DCs and this is thought to be due to the inhibition of viral replication by type I interferons in CD11b+ DCs [19]. Whilst not investigated in these experiments, it is possible that following BTE sensitisation CD11b+ DCs no longer produce type I interferons, allowing them to become permissive to viral replication.

In addition to altered DC migration and function, a subsequent effect was also seen on the CD8 T cell populations in the lungs. Sensitisation prior to infection led to an earlier increase in CD8 T cells and virus specific CD8 T cells in the lungs, which peaked at day 8 p.i., earlier that the peak seen in the PBS infected mice. Not only was the peak earlier, but the magnitude of this increase was also much greater in infected BTE sensitised mice and these T cells were also found to produce more IFN-γ. Interestingly, at later time points after infection (day 14 p.i.) there was a significant decrease in the number of T cells and those producing IFN-γ in the lungs of BTE sensitised and infected mice compared to PBS infected mice, indicating there was faster resolution of influenza virus induced disease and reduced lung damage. In supported of this, we also found a reduction in the amount of albumin leakage in the airways of influenza infected BTE sensitised mice compared to PBS infected mice at day 14 p.i. In contrast to the data shown by Furuya *et al*, we find that T cells play a key role in the enhanced protection to influenza in asthmatic mice. These differences could be due to the use of a different asthma model. In their studies the majority of the experiments were performed using an OVA sensitisation and challenge model, and the time between the last challenge and infection was also much longer compared to our study, where infection was given twenty-four hours after the last allergen challenge. Interestingly, even though they saw a reduction in immunopathology in infected asthmatic mice, they did not see a difference in viral clearance [13]. This again is in contrast to our findings where we show that BTE sensitisation prior to infection had no effect on the replication of the virus in the lung, but did in fact enhance the clearance of the virus, presumably through the increased number and activity of virus specific CD8 T cells.

As well as increasing the numbers of DCs, T cells and eosinophils, BTE sensitisation also increased NK cell numbers in the MLN. A significant increase was also seen in the airways of BTE sensitised mice following influenza infection, but not in PBS infected mice. NK cells have been shown to be important in both the establishment of allergic disease [31] and for the migration of DCs to the MLN following influenza infection [32]. Whilst we did not investigate the role of NK cells in our model, others have studied the effect of depleting NK cells in models of asthma and influenza, but these studies have yielded conflicting results [13, 33]. In addition, we also observed an increase in the presence of mucus secreting goblet cells in the lungs of BTE sensitised and infected mice compared to PBS infected mice. Whilst we did not investigate the role of excess mucus production in the protection against influenza infection, it is possible that the presence of more mucus within the airways led to less of the virus being available to infect the epithelium.

In conclusion, we show for the first time that repeated low dose sensitisation with *B*. *tropicalis*, prior to influenza infection leads to an augmented innate and adaptive immune response in the MLN, lungs and airway. Sensitised and infected mice were found to have increased numbers of CD11b+ DCs in the MLN, which were found to be capable of priming CD8 specific T cells *in vivo* and *ex vivo*, resulting in the earlier appearance of T cells in the lungs, faster clearance of the virus, and subsequently reduced weight loss and reduced lung damage. Whilst the focused of these studies has been on the role of dendritic cells and T cells, the role other immune cells may play in lessening influenza pathology cannot be ruled out and warrant further investigation. However, we believe these studies provide a novel explanation for the protective effects of asthma that were seen in patients during the 2009 pandemic. Several groups including our own have now reported on the phenomenon of asthma being a protective state against influenza induced pathology. Whilst this does not appear to be true for other respiratory viruses, it is a significant discovery which could help in the future treatment of asthmatics during subsequent influenza outbreaks.

## Supporting information

S1 Fig ARRIVE checklist. A completed ARRIVE guidelines checklist is included for all animal studies performed.
(PDF)

S2 Fig Flow cytometry gating strategy for DCs. Mice were sensitized with either PBS or 0.5μg of BTE 3 times a week, for 2 weeks. 24 hr after the last sensitization mice were culled and cellular infiltrate into the MLN and lungs were measured. Representative flow plots are shown for the gating strategy used to identify eosinophils, neutrophils, macrophages, CD103+ and CD11b+ DCs in the MLN and lungs. (A) Live cells were identified first (G1), followed by (B) exclusion of doublets (G2). (C) FSC versus SSC was used to gate around the granulocyte population (G3) and the lymphocyte/DC population (G4). (D) Neutrophils were identifed as Ly6G positive (G5) and (E) esoinophils were identified as Ly6G negative (G6) and SiglecF positive (G7). Macrophages were identified as Ly6G negative (G6) and positive for SiglecF and CD11c (G8) and confirmed to not express CD103 or CD11b (F). (G) Dendritic cells were identified as MHC class II+ high and CD11c+ high cells gated from G4 and then identifed as either (H) CD103+ (G10) or CD11b+ (G11).
(TIF)

S3 Fig Gating strategy for DCs isolated by FACS. Mice were sensitized with either PBS or 0.5μg of BTE 3 times a week, for 2 weeks. 24 hr after the last sensitization mice were infected with 500 PFU of influenza PR8-OVA virus. Mice were culled at day 3 p.i. and the MLN isolated. Representative flow plots are shown for the gating strategy used to sort CD103+ and CD11b+ DCs. (A) and (B) Single live cells were first identified. (C) A FITC dump channel was then used to exclude CD3+, CD4+, CD8+, NK and B cells. (D) MHC class II+ high and CD11c+ high cells were then gated, from which (E) CD103+ and CD11b+ DCs were identified and collected.
(TIF)
