# Supplementary figures and images for: Prior exposure to inhaled allergen enhances anti-viral immunity and T cell priming by dendritic cells
Source: PLoS One. 2018 Jan 2;13(1):e0190063. doi: 10.1371/journal.pone.0190063 (PMC5749744; doi:10.1371/journal.pone.0190063)

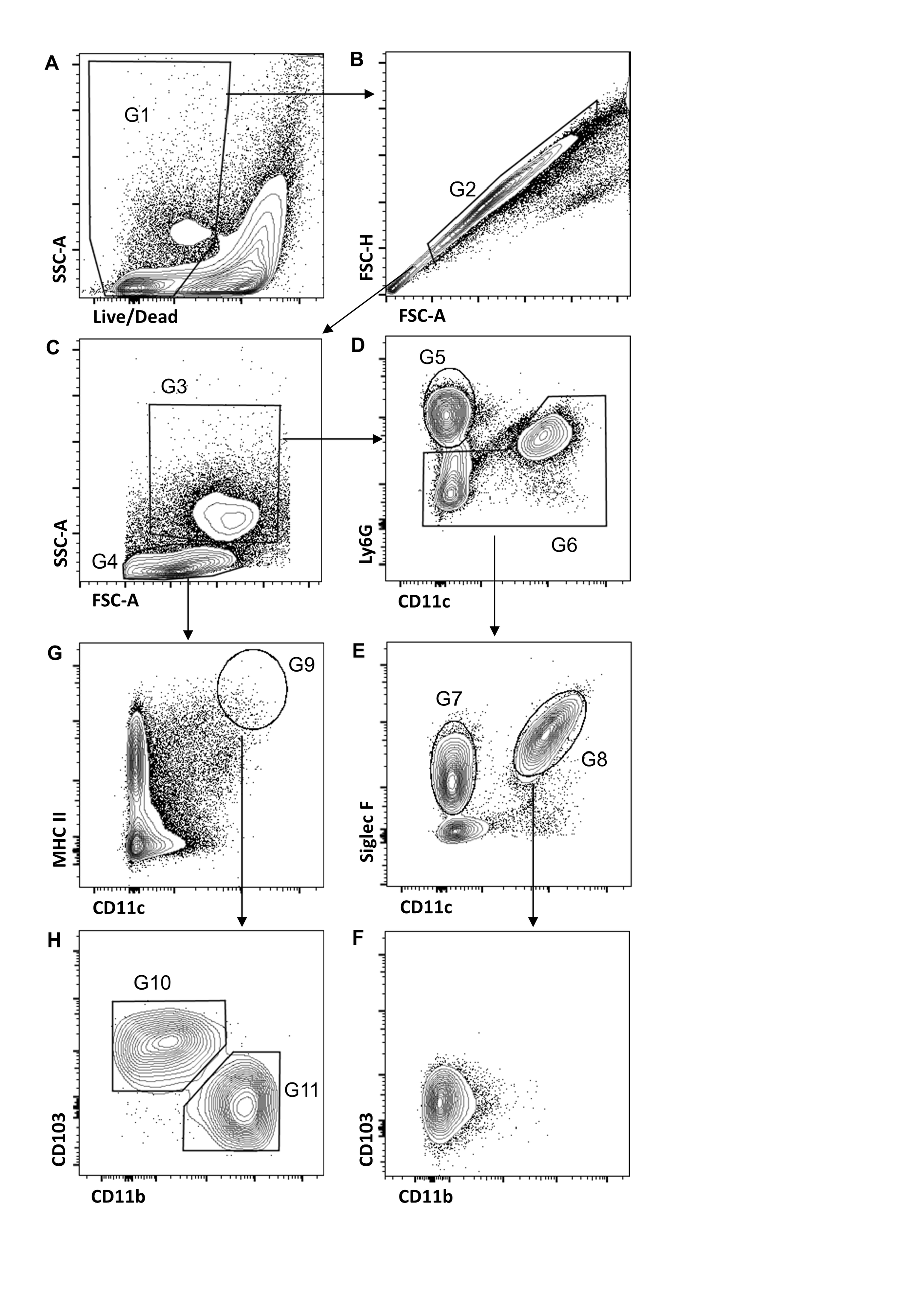

Supplement: S2 Fig — Mice were sensitized with either PBS or 0.5μg of BTE 3 times a week, for 2 weeks. 24 hr after the last sensitization mice were culled and cellular infiltrate into the MLN and lungs were measured. Representative flow plots are shown for the gating strategy used to identify eosinophils, neutrophils, macrophages, CD103+ and CD11b+ DCs in the MLN and lungs. (A) Live cells were identified first (G1), followed by (B) exclusion of doublets (G2). (C) FSC versus SSC was used to gate around the granulocyte population (G3) and the lymphocyte/DC population (G4). (D) Neutrophils were identifed as Ly6G positive (G5) and (E) esoinophils were identified as Ly6G negative (G6) and SiglecF positive (G7). Macrophages were identified as Ly6G negative (G6) and positive for SiglecF and CD11c (G8) and confirmed to not express CD103 or CD11b (F). (G) Dendritic cells were identified as MHC class II+ high and CD11c+ high cells gated from G4 and then identifed as either (H) CD103+ (G10) or CD11b+ (G11). (TIF) [file pone.0190063.s002.tif]

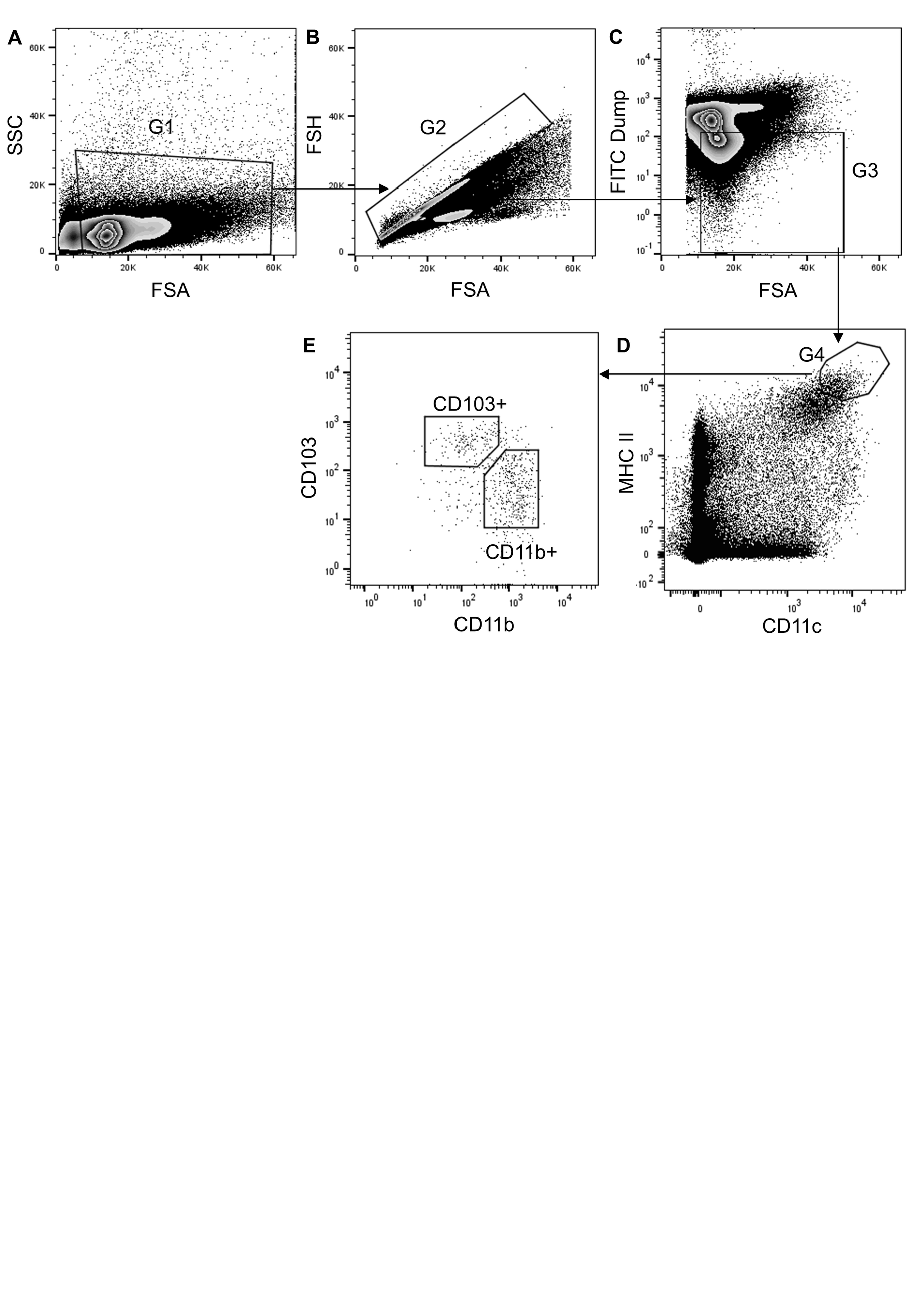

Supplement: S3 Fig — Mice were sensitized with either PBS or 0.5μg of BTE 3 times a week, for 2 weeks. 24 hr after the last sensitization mice were infected with 500 PFU of influenza PR8-OVA virus. Mice were culled at day 3 p.i. and the MLN isolated. Representative flow plots are shown for the gating strategy used to sort CD103+ and CD11b+ DCs. (A) and (B) Single live cells were first identified. (C) A FITC dump channel was then used to exclude CD3+, CD4+, CD8+, NK and B cells. (D) MHC class II+ high and CD11c+ high cells were then gated, from which (E) CD103+ and CD11b+ DCs were identified and collected. (TIF) [file pone.0190063.s003.tif]
